# Study protocol: The registrar clinical encounters in training (ReCEnT) study

**DOI:** 10.1186/1471-2296-13-50

**Published:** 2012-06-06

**Authors:** Simon Morgan, Parker J Magin, Kim M Henderson, Susan M Goode, John Scott, Steven J Bowe, Catherine M Regan, Kevin P Sweeney, Julian Jackel, Mieke L van Driel

**Affiliations:** 1 General Practice Training-Valley to Coast, Gavey St, Mayfield, 2304, NSW, Australia; 2 Discipline of General Practice, Newbolds Building, University of Newcastle, Callaghan, 2308, NSW, Australia; 3 Research Centre for Gender, Health and Ageing, University of Newcastle, Callaghan, 2308, NSW, Australia; 4 Bridge Medical Centre, Crawley, West Sussex, RH117BQ, UK; 5 Faculty of Health Sciences and Medicine, Bond University, Queensland, Australia; 6 Department of General Practice and Primary Health Care, Ghent University, Ghent, Belgium; 7 Discipline of General Practice, School of Medicine, University of Queensland, Brisbane, 4009, Australia

## Abstract

**Background:**

Patient encounters are the core learning activity of Australian general practice (family practice) training. Exposure to patient demographics and presentations may vary from one general practice registrar (vocational trainee) to another. This can affect comprehensiveness of training. Currently, there is no mechanism to systematically capture the content of GP registrar consultations. The aim of the Registrar Clinical Encounters in Training (ReCEnT) study is to document longitudinally the nature and associations of consultation-based clinical and educational experiences of general practice registrars.

**Methods/design:**

This is an ongoing prospective multi-site cohort study of general practice registrars’ consultations, entailing paper-based recording of consultation data. The study setting is general practices affiliated with three geographically-based Australian general practice regional training providers. Registrars record details of 60 consecutive consultations. Data collected includes registrar demographics, details of the consultation, patient demographics, reasons for encounter and problems managed. Problems managed are coded with the International Classification of Primary Care (second edition) classification system. Additionally, registrars record educational factors related to the encounter. The study will follow the clinical exposure of each registrar six-monthly over the 18 months to two years (full-time equivalent) of their general practice training program.

**Conclusions:**

The study will provide data on a range of factors (patient, registrar and consultation factors). This data will be used to inform a range of educational decisions as well as being used to answer educational research questions. We plan to use ReCEnT as a formative assessment tool for registrars and help identify and address educational needs. The study will facilitate program evaluation by the participating training providers and thus improve articulation of educational programs with practice experience. From the research point of view it will address an evidence gap – the in-practice clinical and educational experience of general practice trainees, determinants of these experiences, and the determinants of registrars’ patterns of practice (for example, prescribing practice) over the course of their training.

## Background

Consulting with patients is the core learning activity of general practice (family practice) training in Australia. Registrars (general practice vocational trainees) learn by the “apprenticeship model”, seeing patients in the general practice setting under the supervision of accredited general practitioner (GP) supervisors. Ideally, the content of each registrar’s clinical experience should include “common and significant conditions” [1] and be similar to that of non-trainee (established) Australian GPs, as reflected in the curricula of the Royal Australian College of General Practitioners (RACGP) [2] and the Australian College of Rural and Remote Medicine (ACRRM) [3]. Indeed, the development of sound clinical reasoning skills appears to be dependent on exposure to ‘an adequate database’ of clinical cases [4].

However, in real life, the curriculum “walks through the door”, and anecdotally, the exposure to different patient demographics and presentations is highly variable between training practices and between one registrar and another. This variability is likely to have an impact on the comprehensiveness and quality of training.

The content of clinical encounters in general practice have been described in studies from a number of countries [5-7], including the BEACH (Bettering the Evaluation and Care of Health) program in Australia [8]. Other Australian studies have specifically looked at patient encounters in the Aboriginal Medical Service context [9-11] and between urban and rural settings [12].

Reports of clinical encounters of registrars in general practice training are scarce. Despite the importance of the area both clinically and educationally, to date there have been no peer-reviewed publications on the content and nature of patient encounters with registrars in Australian general practice training.

The ReCEnT (Registrar Clinical Encounters in Training) study aims to longitudinally document the nature and associations of consultation-based clinical and educational experiences of general practice registrars. In particular, it aims to identify variability in the clinical exposure between individual registrars, and explore associations of such variability. It will also establish the determinants, including clinical and educational experiences during training, of registrars’ patterns of practice (for example, prescribing practice) by the conclusion of their training.

## Methods

### Study design

The ReCEnT study is an ongoing prospective cohort study.

### Study aims

The study aims to document multiple factors (registrar, practice, patient, encounter, clinical, educational) in registrars’ clinical consultations and to establish associations of these factors. The study will establish determinants of a number of outcomes involving registrars’ clinical and educational activity.

Initial broad hypotheses to be tested include, that

● The demographics of patients seen by GP registrars in consultations (including age, sex, socioeconomic status (SES), Indigenous status, language other than English spoken, measure of rurality of residence) and the patient diagnoses/problems managed in consultations, will be associated with registrar factors (including age, sex, language other than English spoken, country of graduation, prior medical experience) and practice factors (including size of practice, measure of rurality of location).

● Consultation factors (including duration of consultation, number of problems dealt with, medication prescribed, type of billing, pathology tests ordered, imaging studies ordered and referrals made, and occurrence of violence within the consultation) will be associated with the above patient, registrar and practice factors.

● Educational outcome factors (including recourse to advice from the registrars’ supervisors or other senior clinicians, use of hard-copy or electronic sources of information, and generation of learning goals) will be associated with the above registrar, practice, and patient factors.

### Setting

The Australian General Practice Training (AGPT) program is responsible for administering the vocational training for general practice in Australia [13]. This training is regionalised, with delivery of training devolved to seventeen regional training providers (RTPs) around the country. Individual RTPs co-ordinate registrar training and provide discrete educational activities. However, the majority of registrar training activities occur in general practices, accredited and supported by their local RTP, rather than RTP educational activities.

The AGPT involves a minimum of two years (full-time equivalent) training post hospital experience. Minimum requirements for completion of training are three 6-month terms in general practice, and a further 6-month term in general practice or another discipline.

This is a multi-site study. The setting of the study is the accredited practices of three RTPs. The individual RTPs encompass major city and inner regional [14] practices (in the state of New South Wales), major city practices (in Victoria) and inner and outer regional and remote practices (in Tasmania).

### Participants

All registrars of the three participating RTPs who are undertaking general practice terms participate. As well, registrars training in community-based, non-general practice positions participate. These include posts in dermatology, family planning, community psychiatry and the Prevocational General Practice Placement Program (PGPPP), where doctors training in the hospital setting undergo a specific placement in general practice [15].

### Recruitment

Registrars are recruited by direct contact at regular educational release workshops at the three participating RTPs. Participation in ReCEnT is part of registrars’ training requirements. Registrars also have the option of consenting to their data being used for research purposes (via an ‘opt-in’ consent process for the research aspect of the project).

### Data collected

Variables for which data is collected can be considered as the registrar, practice, patient, encounter, clinical, educational and occupational violence factors.

*Registrar variables:* Registrars’ demographics, past educational and work experience and current training term as detailed in Additional file 1: Table S1.

*Practice variables:* Factors such as practice size, remoteness classification and socioeconomic status of the practice location as detailed in Additional file 1: Table S2.

*Patient variables:* Patient demographics and reasons for encounter (RFE) as per Additional file 1: Table S3.

*Encounter variables*: Factors such as date of consultation, consultation duration and type of billing as per Additional file 1: Table S4.

*Clinical variables*: Factors such as problems managed, procedures performed, investigations ordered and follow-up arranged as per Additional file 1: Table S5.

*Educational variables*: A range of educational factors related to the consultation as per Additional file 1: Table S6.

*Exposure to violence*: In addition, registrars will answer a question about exposure to violence during the consultation. If they have been exposed to violence, they will record details of the episode (nature of the violence as per the classification system of Tolhurst et al. [16] modified by Magin et al. [17], and the location and precipitants of the violence).

### Data collection instrument

A paper-based data collection instrument was developed, based on the BEACH study tool and patient encounter tools from similar studies [5,6].

### Data collection

Registrars undertake formal orientation and training in the ReCEnT study during a dedicated face-to-face orientation workshop. This comprises the background and rationale of the study and information on how to complete the patient encounter forms. Registrars who are unable to attend group workshops are given individual detailing on the project.

Registrars record the details of sixty consecutive patient encounters at the mid-point of their six month general practice training terms (for full-time registrars generally April and October). This represents approximately one week of consultations for a full-time first term registrar. Registrars record only consultations conducted in the general practice setting (that is, not those conducted in a nursing home, or on a home visit). Consultations conducted as part of a specialised clinic (e.g. immunisation or anticoagulation management) are also excluded.

### Data coding

Data is entered into a Microsoft Access database. Data on reason for encounter, problems managed, investigations and referrals made are classified using the International Classification of Primary Care, second edition (ICPC2-plus) disease classification system [18].

Medication data is coded using the Anatomical Therapeutic Chemical (ATC) Classification system which has been maintained by the WHO (World Health Organisation) since the 1970s [19].

Data on procedures is coded using a list of procedures relevant to the Australian general practice context derived by means of a Delphi process prior to the study [20].

### Data analysis

Simple descriptive statistics (mean, median, proportion, 95% confidence intervals, standard deviation etc.) will be used to describe the various registrar, practice, patient, encounter, clinical, educational and occupational violence parameters. Student t-tests (or non-parametric equivalent) or chi-square analyses, as appropriate, will be used to make univariate comparisons of registrar and practice groups on these outcomes. Further multivariable analyses of these outcomes will employ multiple linear regression or logistic regression as appropriate.

Random Effects modeling and Generalised Estimating Equations (GEE) adjusting for clustering at RTP, practice and registrar level will be employed in analysing changes over time in the relevant clinical and educational outcomes.

The Bonferroni adjustment will be made for multiple comparisons and multiple outcomes for those analyses in which it is appropriate.

### Ethics

Ethics approval for the study was obtained from the Human Research Ethics Committee, University of Newcastle. Approval number: H-2009-0323.

### Pilot study

A pilot was conducted in late 2009 in which 32 registrars from a single RTP returned patient encounter forms for 1919 consultations. Following data collection and entry, a questionnaire was completed by participating registrars eliciting opinions concerning all aspects of the process, especially the time taken to complete the data collection, effect of the data collection on clinical practice, difficulties with interpretation of the study instructions or difficulties in recording aspects of the consultation, and ease of use of the study data collection instrument. A focus group with five of the participating registrars was conducted to further explore the same issues. Based on this pilot study and qualitative evaluation a number of minor changes were made to the study data collection form and data collection processes.

## Discussion

### ReCEnT in the context of previous patient encounter studies and the use of logbooks in training

Australian vocational training programs across a number of disciplines employ log book systems to record details of (generally non-consecutive) clinical and procedural encounters e.g. psychiatry [21], medicine [22], and surgery [23]. However, there is no national requirement for completion of a patient log, or indeed any systematic recording of consultation details, in the Australian General Practice Training program.

Logbooks of non-consecutive patient encounters are unlikely to adequately reflect the breadth and composition of registrars’ clinical experience. Studies of consecutive patient encounters are better suited to this objective. A British study from 1986 used a database of routinely collected consultation data to show that registrars (compared to GP principals) saw more children and fewer elderly patients, more patients with acute respiratory disorders, fewer patients presenting for preventive care and fewer patients with cardiovascular disorders [24]. There were also differences in prescribing and referral patterns. A more recent Dutch study using prospectively recorded consultation data described a number of factors that influenced clinical exposure, including trainee and practice issues [25].

Australian GPs have been periodically recording the nature and content of their consultations since the first national survey of morbidity in 1961 [26]. In 1990, the first large and comprehensive national survey of morbidity and its management in general practice (the Australian Morbidity and Treatment Survey, AMTS) was conducted [27]. The study was the first to use a stratified random sample of general practitioners and provided extensive information on the content of over 110,000 consultations in Australian general practice.

Since 1998, the BEACH (Bettering the Evaluation and Care of Health) program, coordinated by the Family Medicine Research Centre at the University of Sydney, has annually enrolled a random sample of 1000 GPs, each of whom prospectively records the details of 100 consecutive patient encounters [28].

However, none of these previous Australian studies have specifically looked at GP registrar activity and all have employed cross-sectional methodology.

The ReCEnT study differs from previous research into general practice clinical activity in a number of key areas. Firstly, it directly targets general practice registrars training in the Australian context. As a result, the study will have a specific focus on linking clinical exposure to educational needs. Furthermore, the ReCEnT study is longitudinal, following the clinical exposure and clinical practice of the same participants over at least 18 months and at least three data points. It will thus provide a description of temporal changes in registrars’ clinical experiences and practice, and enable us to establish determinants of these experiences and of registrars’ patterns of practice (for example, prescribing practice) over the course of their training.

This will also be the first general practice study to look at the educational aspects of routine clinical practice. Registrars are a particularly appropriate group in which to examine the effects of clinical experience and educational factors on clinical practice, having less established patterns of practice than more experienced GPs. The longitudinal nature of the study will enable us to make some inference regarding causality of such patterns of practice. The study will also provide a framework for trialing educational interventions in this early-career, educationally-receptive cohort (see below).

### Choice of data collection methodology

Log books have been used in the undergraduate setting for many decades. These have comprised a wide variety of formats, including handheld (e.g. pocket-sized encounter cards [29]), optically scanned [30] and electronic (PDA [31], web-based [32]). Though electronic formats have become increasingly accessible, there is little evidence that this format improves accuracy or completeness of data collection [33].

The ReCEnT study employs a paper based, self-reported data collection system. Though this poses some limitations, there are practical reasons why this methodology was chosen.

Extraction of routinely collected electronic data from general practice settings for research purposes occurs routinely in many countries, including the UK and Netherlands [34,35]. However, due to the large and diverse variety of software packages in Australian general practice, efficient extraction of routinely collected electronic data is currently impossible. Furthermore, routinely recorded data in Australian general practice is also likely to be of relatively poor quality compared to deliberately collected records. There is evidence that data obtained specifically from encounter forms is more comprehensive and more reliably coded than that obtained from medical records in the Australian general practice setting [36]. Also, much of the study data would not be recorded routinely in clinical records, including the educational aspects (seeking of advice, formulating learning goals) and experience of occupational violence.

The self-reported method of data collection does pose a risk of reporting bias. However, we have attempted to minimise reporting bias in a number of ways. Registrars (and their practices and clinical supervisors) are educated extensively about the rationale for and procedures of the study. Practices ‘rule off’ consultation appointments in each session in which ReCEnT data collection takes place as ‘catch up’ for the time involved in completion of data collection forms (which averages less than two minutes each form). The importance of recording consecutive (not selected) consultations is heavily emphasised.

Registrars are required to record the details of sixty consecutive patient encounters per general practice training term, equivalent to approximately one week of consultations for a full-time first term registrar. This figure was derived from a number of considerations. Primarily, we believe this is a reasonable balance between registrar acceptability (time taken to complete forms) and representativeness of clinical and educational exposure. The BEACH study requires participating GPs to record 100 encounters – however, qualified GPs have shorter consultations than registrars and less educational requirements built into their working week. A further consideration was that over the duration of vocational training, registrars will record a minimum of 180 patient encounters across three terms, with many recording further data in optional training terms (up to a total of 240).

### Coding and classification

BEACH data is classified using the ICPC-2PLUS coding system, the international standard for classifying primary care data. The validity of this system has previously been demonstrated [37]. Furthermore, there is evidence of close convergence between GP and patient recording of reasons for encounter (RFE) and problems managed in a consultation [38] and the reliability of secondary coding of RFEs [39]. The ReCEnT study employs ICPC2-plus as a classification system, and therefore data will be comparable with that of the BEACH study.

The ATC medication database is the Australian standard for classifying medications at the generic level, and is a tool for medication utilisation research in many other countries [19]. The hierarchical structure of the classification system contains five levels which reflect the organ or body system on which the drug acts and its therapeutic, pharmacological and chemical characteristics.

### Planned and potential applications of ReCEnT data

General practice clinical activity data has a vast range of realised and potential applications, including workforce planning [40,41], health service planning and policy development [42], quality improvement [43], monitoring of patient safety [44], population health and community need assessment [45-47], and educational planning and development. The latter is the explicit focus of the ReCEnT study and is arguably the least well explored in previous research.

#### *Applications for the individual registrar learner*

In relation to individual student education, logbooks have primarily been used as tools for reflection and feedback [48], and to measure achievement of educational objectives [49]. In addition to standard consultation data, log books have been used to track student-generated learning needs arising from the clinical encounter and apply these directly to education [50].

The patient encounter data from the ReCEnT study, as with undergraduates, acts as a mechanism for registrars’ formative assessment, allowing them to reflect on their practice in comparison to peers and to other benchmarks. Registrars are given a feedback report after each round of data collection, detailing their individual data and comparing this to aggregate registrar data and their previous round data. Using this report, registrars are able to make broad observations about curriculum coverage (demographics, complexity, acuity, continuity) and identify gaps in clinical exposure. Identification of registrar exposure to specific clinical presentations is limited by the modest number of encounters documented, but broad observations about coverage e.g. Women’s health, ENT etc. is possible. Learning needs or identified clinical gaps can then be addressed with a variety of educational interventions, for example self-directed study, targeted tutorials, planned clinical placements, and targeted patient booking.

Registrars are able to compare their individual data with indicators of best practice, for example for prescribing patterns, as well as reflect on any change in practice towards best practice benchmarks over training time. This process will also provide them with essential skills for life long learning.

Portfolios are widely used in general practice training and are effective in encouraging reflective learning [51]. However, there is no published literature on the use of a patient log to stimulate encounter-based learning needs in general practice training. Similarly, there is no literature on the use of a patient encounter log to compare registrar performance with indicators of evidence-based or best practice, for example, antibiotic prescribing rates [52]. The ReCEnT study aims to directly address these evidence gaps.

#### *Applications for program evaluation and quality improvement*

Patient encounter data has been used extensively by medical schools for program evaluation and curriculum review [33]. A key aspect of general practice training delivery is program evaluation and quality assurance and improvement. The RACGP vocational training standards state in their recommendations for quality improvement that the regional training provider (RTP) should provide evidence of the effectiveness of the educational processes employed [1].

The ReCEnT study data will provide information on curriculum coverage and registrar performance across the registrar cohort. Patient encounter data will help identify differences in learning opportunities across different practices in the training region, as well as those across different models of service delivery, for example, private practices and Aboriginal Medical Services. This information will help tailor educational programs for individual registrars (as has been used in the undergraduate setting [53]), and allow better articulation of RTP educational release activities with practice experience.

Further potential applications of the study in relation to program evaluation are to evaluate the clinical exposure and fulfillment of specific learning goals within specific types of terms (for example, urban versus rural posts, and ‘usual’ general practice terms as opposed to extended skill posts, Family Planning, remediation terms etc.). As well, ReCEnT data will facilitate assessment of effectiveness of educational interventions, for example registrar practice before and after a workshop session.

In addition, patient encounter data can help support the teaching and supervision role of the GP supervisor. Aggregated clinical exposure and educational data can help identify the strengths and weaknesses of individual practices, and better inform practice-based teaching and other educational interventions e.g. targeted patient booking.

Within Australia, we believe that the generalisability of the study findings in relation to program evaluation will be high. The Australian general practice training program is a national program with closely prescribed (and enforced) procedures and standards across all Regional Training Providers.

##### *Research applications*

The other major application of the ReCEnT study is that of research. The RACGP vocational training standards state in their recommendations for quality improvement that registrars should have the opportunity to undertake elective research [1]. The ReCEnT study will provide a platform for quality registrar research activities, and therefore build research capacity in general practice registrars. This is particularly the case with registrars wishing to undertake Academic Extended Skills posts (an optional component of vocational GP training in Australia).

As well as registrar-initiated research, the study is expected to be the vehicle for a diverse range of other research. In particular, the longitudinal methodology of the study will provide scope for enquiry in a number of areas.

The ReCEnT study will address a particular evidence gap – the clinical and educational experience of general practice trainees, the determinants of these experiences, and the determinants of registrars’ patterns of practice (for example, prescribing behaviour) over the course of their training. Findings in these areas will inform general practice in Australia beyond the participating RTPs and will have implications for GP training programs internationally.

Similarly, establishing patterns of registrars’ ‘help- and advice-seeking’ behaviour and its association with prescribing and other management decisions will inform models of supervision and teaching of ‘problem-based’ and ‘self-directed’ learning.

As well as these applications of the central descriptive study, ReCEnT provides a platform for trials of educational interventions. An example would be the effectiveness of an educational intervention promoting rational prescribing in producing desirable changes in registrars’ prescribing practice.

Occupational violence is a major issue in general practice in Australia and internationally, with effects both on the individual and their provision of services to patients [54,55]. There is some evidence that GP registrars, particularly rural registrars, are at particular risk of occupational violence and its effects [56]. A major limitation of all previous research of this problem has been it’s cross-sectional methodology, relying on GPs’ recall of incidents. The ReCEnT study is the first prospective study of occupational violence in general practice.

## Conclusion

The ReCEnT study will provide comprehensive information on the clinical exposure of Australian GP registrars. In addition, with its specific focus on educational needs, it will provide novel reflective and evaluative data for registrars and RTPs and will build the research capacity of GP registrars and Australian general practice.

## Competing interests

The authors declare that they have no competing interests.

## Authors’ contributions

SM and PM led the design of the study. SM led the writing of the manuscript. All other authors contributed to the study design and have all contributed to the final manuscript. All authors read and approved the final manuscript.

## Pre-publication history

The pre-publication history for this paper can be accessed here:

http://www.biomedcentral.com/1471-2296/13/50/prepub

## Supplementary Material

Additional file 1 **Table S1.** Registrar Variables. **Table S2.** Practice Variables. **Table S3.** Patient Variables. **Table S4.** Encounter Variables. **Table S5.** Clinical Variables. **Table S6.** Educational Variables. Click here for file
