# Supplementary material for: Study protocol: The registrar clinical encounters in training (ReCEnT) study
Source: BMC Fam Pract. 2012 Jun 6;13:50. doi: 10.1186/1471-2296-13-50 (PMC3507666; doi:10.1186/1471-2296-13-50)
Supplement: Additional file 1 — Table S1. Registrar Variables. Table S2. Practice Variables. Table S3. Patient Variables. Table S4. Encounter Variables. Table S5. Clinical Variables. Table S6. Educational Variables. [file 1471-2296-13-50-S1.doc]

**Table 1. Registrar Variables**

| - Gender - Date of birth - Training term - Full-time/part-time status - Country of birth - Main language spoken - Prior University qualifications in a health-related field - Prior University qualifications in a non health-related field - Country where primary medical degree obtained - Year of graduation - Years working in a hospital post-internship - Post-graduate qualifications in medicine - Auspicing College (RACGP ^1^ or ACRRM ^2^) - GP Pathway enrolled in - Previous mental health training and attitudes to mental illness |
| --- |

1. Royal Australian College of General Practitioners

2. Australian College of Rural and Remote Medicine

**Table 2. Practice Variables**

| - Size of Practice - Remoteness classification - Socioeconomic status of the Practice - Number of Practice nurses - Practice billing procedure - Internet access - Computer use within Practice |
| --- |

Table 3. Patient Variables

| - Date of birth - Gender - Postcode - Non-English speaking background (NESB) - Aboriginal/Torres Strait Islander |
| --- |

**Table 4. Encounter Variables**

| - Date of consultation - Duration of consultation - Type of billing - New patient to Practice - New patient to registrar - Seen previously in the recording period - Medicare item numbers |
| --- |

Table 5. Clinical Variables

| - Reasons for encounter - Problems managed - New or old Problem - Dr initiated problem - Procedures performed - Medications prescribed - Investigations made (pathology, imaging/other tests) - Use of Practice Nurse - Referrals - Follow-up arranged |
| --- |

**Table 6. Educational Variables**

| - Sources of assistance for patient care during the consultation:   - Supervisor/other Dr in Practice   - Specialist   - Other Health Professional   - Electronic resources   - Books   - Other sources - Learning goals generated |
| --- |
